# Developing a Program Evaluation Training Within a National Federal Healthcare Setting: Veterans Affairs Evaluation Bootcamp Training

**DOI:** 10.1002/lrh2.70102

**Published:** 2026-07-13

**Authors:** Nardeen M. Shafik, John P. Donnelly, Jessica Johnson, Linda M. Kawentel, Nicholas W. Bowersox

**Affiliations:** ^1^ VA QUERI Center for Evaluation and Implementation Resources Ann Arbor Michigan USA; ^2^ VA HSR Center for Clinical Management Research Ann Arbor Michigan USA; ^3^ Department of Learning Health Sciences University of Michigan Ann Arbor Michigan USA

**Keywords:** application of evaluation skills, evaluation capacity building, evaluation skills development, learning health systems, virtual training design

## Abstract

**Introduction:**

Learning Health Systems (LHSs) depend on the ability to effectively evaluate programs and translate data into practice. Within the Veterans Affairs (VA) health system, formal mandates require programmatic decision‐making to be data‐driven; however, opportunities for staff to develop applied program evaluation skills that support LHS learning cycles remain limited.

**Methods:**

To address this need, the synchronous virtual Evaluation Bootcamp Training (EBcT) was developed to equip learners with practical, applied evaluation skills. This manuscript describes the design rationale, structure, and core concepts of the training, emphasizing design decisions intended to support evaluation capacity building within a large and complex health system.

**Results:**

EBcT was delivered over 4 days across 2 training cohorts (8 learners in 4 project teams from Cohort 1; 12 learners in 5 project teams from Cohort 2). Project teams addressed a range of clinical and operational initiatives within VA. Learners represented diverse professional roles, including lead evaluators, clinical subject matter experts, project managers, operational leaders, and analysts. Learners reported high satisfaction throughout the training, with full attendance at application sessions and strong participation in didactic components. A majority reported meeting predefined evaluation learning targets at the end of the training.

**Conclusion:**

Findings suggest that structured, team‐based training models such as EBcT can strengthen evaluation capacity and support LHS functioning by equipping project teams to engage in repeated cycles of measurement, learning, and performance improvement.

## 1 Introduction

Program evaluation is an essential component of healthcare, particularly within the context of Learning Health Systems (LHSs) [1]. LHSs provide a structured framework for continuous learning where data‐driven decisions guide the improvement of clinical practices and organizational processes. This integration of evaluation and application allows for real‐time refinements to healthcare practices, ensuring their effectiveness and sustainability and enabling the creation of strategic goals based on specific targets for improvement [2]. Enhancing the evaluation skills of healthcare employees serves to promote maturity for developing LHSs and improve the capability of organizations to provide infrastructural services that are key for effective LHS functioning [3, 4].

The U.S. Department of Veterans Affairs (VA) healthcare system serves as one of the nation's largest and most advanced LHS. By embedding program evaluation into healthcare practice, VA enhances organizational effectiveness and ensures that evidence‐based decision‐making drives clinical operational management consistent with formal mandates (e.g., H.R.4174—115th Congress, 2019) [5]. To further support internal evaluation efforts, VA has developed formal guidance materials designed to provide direction to persons interested in conducting program evaluation activities based on best practice recommendations and consistent with formal expectations for quality improvement initiatives [6].

Although formal guidance on how to conduct program evaluation within VA is available, gaps remain in the workforce's capacity to effectively engage in program evaluation activities which include steps related to evaluation design, data collection and analysis, interpretation of results, and application of program evaluation findings to support application within repeated learning cycles [7]. To address this gap, the VA Quality Enhancement Research Initiative (QUERI) Center for Evaluation and Implementation Resources (CEIR) created the Evaluation Bootcamp Training (EBcT) [8, 9]. EBcT is designed to expand VA program evaluation capacity by educating learners on the processes involved in designing and implementing evaluations, increasing awareness of VA evaluation expectations, and encouraging strategic thinking about opportunities to strengthen evaluation practices. This team‐focused training requires learners to bring real‐world evaluation projects to the training, enabling practical application of learning concepts in a concrete manner that can be directly translated into action. By focusing on applied training in program evaluation best practices, EBcT assists VA employees in developing skills which allow them to design and conduct applied, impactful program evaluations.

This manuscript describes the design rationale, structure, and core concepts included in EBcT, and includes initial findings based on formative feedback from the first two EBcT training cohorts. It also illustrates how structured evaluation training can support evaluation capacity building as a foundational enabler of LHS performance. As far as the authors are aware, this is the first manuscript describing the development of an applied training in program evaluation foundational skills and the ways that such a training can support broader LHS functioning within a large, complex healthcare system.

## 2 Methods

### 2.1 EBcT Training Development

EBcT development started via the efforts of a workgroup comprised of VA program evaluation experts across multiple VA evaluation centers. The term “Bootcamp” was intentionally selected to reflect the VA's strong cultural ties to military training traditions, where “boot camp” refers to an intensive learning experience designed to build foundational skills and prepare learners for future practice. The EBcT development workgroup assisted in refining the training goals, identifying core evaluation competencies for targeting, and providing recommendations regarding the structure, pacing, and delivery of the training to ensure that content was applied and directly relevant to the work of varied groups. Based on workgroup consensus, the EBcT training curriculum was developed with several learning objectives: increase learners' practical understanding of program evaluation best practices, support strategic thinking about evaluation design and management, and increase familiarity with tools designed to support the direct application of evaluation best practices as endorsed by VA leadership for use in quality improvement projects.

Building from these learning objectives, the EBcT development team then consulted with VA operational offices with experience in conducting national virtual education programs designed to support the development of applied quality improvement skills. These offices routinely coordinate multiday trainings for VA staff located across multiple medical centers and program offices nationwide. EBcT workgroup discussions with these representatives focused on practical considerations related to the design of the EBcT training related to training structure, including pacing across multiple days, maintaining engagement in a virtual format, and integrating project‐based work within a shared learning environment. Based on feedback from this group, the EBcT workgroup elected to structure EBcT in a format that alternated between synchronous didactic instruction and team‐specific facilitated breakout application periods where project teams could directly apply evaluation concepts to their specific projects. This structure was designed to allow for initial foundational instruction followed by immediate application of concepts with expert oversight. This structure was expected to support the rapid development of applied program evaluation technical skills.

**FIGURE 1:**
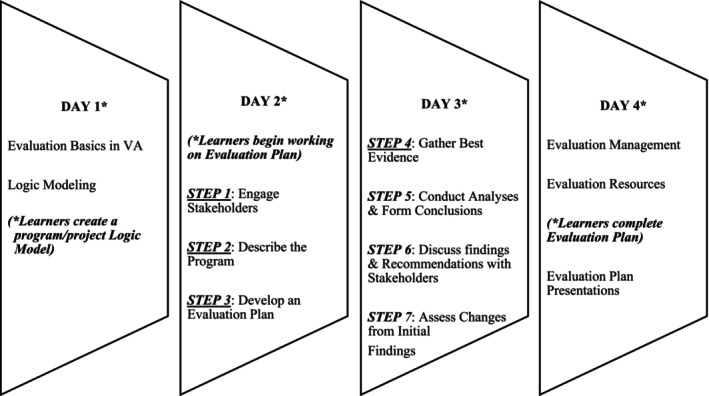
Applied structure of the evaluation bootcamp training. *Breakout Rooms for application after each topic area, and optional office hours offered for support.

EBcT content was designed based on material from the QUERI Evaluation Guide [10], a best‐practice resource which adapts the Centers for Disease Control and Prevention Framework for Program Evaluation in Public Health [8] into a seven‐step evaluation process for application within VA. The QUERI Evaluation Guide has been formally endorsed by QUERI and promoted for broad adoption by VA personnel engaging in quality improvement projects. The QUERI Evaluation Guide seven‐step evaluation development process consists of steps that include engaging stakeholders, describing the program and problem, developing an evaluation plan, gathering best available evidence to assess evaluation questions, conducting analyzes and forming conclusions, discussing findings and recommendations with stakeholders, and assessing changes from evaluation findings. The evaluation process described within the QUERI Evaluation Guide emphasizes iteration and continuity across stages so that early planning decisions shape later analytic and reporting activities and vice versa. This seven‐step evaluation development process served as the organizing backbone of the EBcT training structure. Each step of the QUERI Evaluation Guide framework was translated into instructional modules and applied exercises were developed to support the direct application of core concepts within learners' evaluation projects. Instructional content was translated into structured presentations, templates, and workbooks to ensure project teams produced tangible evaluation products aligned with each stage of the process (Figure 1).

### 2.2 EBcT Training Delivery

EBcT was structured as a 4‐day, partial‐day synchronous virtual program to provide sufficient time for applied instruction while accommodating the scheduling realities of busy VA employees. Training activities included a pretraining kickoff meeting, 4 days of mixed didactics and breakout application sessions, the development of an evaluation plan presentation which was shared with the overall training cohort for feedback, and Daily Feedback Surveys. Each project team was assigned an expert facilitator who worked with them throughout the training.

Prior to each EBcT training, a kickoff meeting was held that introduced speakers, facilitators, and participating project teams and reviewed expectations, timelines, deliverables, and participation requirements. Learners received the QUERI Evaluation Guide, a training manual outlining roles and responsibilities, a structured workbook, and templates including an evaluation plan template and logic model template aligned with the seven‐step framework used during the training. A Microsoft Teams workspace was established to facilitate communication, resource sharing, and document exchange during and after the training.

EBcT didactic content was delivered by VA evaluation experts with experience conducting applied evaluations in collaboration with VA operational partners. EBcT facilitators were experienced evaluators selected based on experience in conducting program evaluations within VA and matched to project teams based on relevant clinical subject matter expertise. Prior to each training, facilitators reviewed Needs Assessment Surveys submitted by participating project teams to understand learner needs, project scope, data availability, and anticipated challenges. During breakout sessions, facilitators worked with project teams to refine evaluation questions, clarify program descriptions, identify feasible data sources, align measures with evaluation questions, and develop structured evaluation plans by reviewing content consistent with the QUERI Evaluation Guide seven‐step evaluation development process.

### 2.3 EBcT Training Evaluation

To assess how well EBcT met its learning objectives, we collected data from project teams at two time periods during their participation in EBcT: at the end of each training day and immediately after completion of EBcT. EBcT evaluation questions development was informed by the Kirkpatrick Model, a widely used framework that assesses training effectiveness across four levels: reaction, learning, behavior, and results [11]. Given the immediacy of the feedback collected from EBcT project teams, evaluation targets initially focused on learner reactions, perceived learning, and anticipated application of evaluation skills, with future evaluation efforts intended to assess results. Surveys were created via an internal iterative development process based on feedback from program evaluation experts who also served as EBcT facilitators and presenters. Following each training day, learners completed brief Daily Feedback Surveys assessing satisfaction.

**FIGURE 2:**
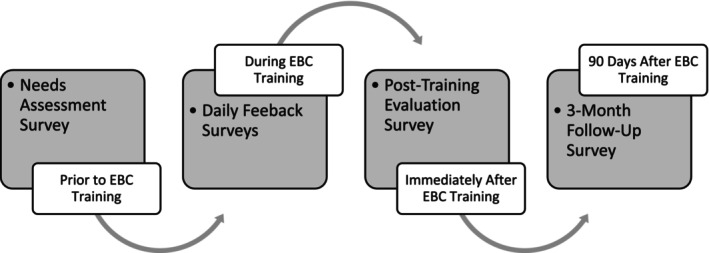
Data collection timeline across the evaluation bootcamp training phases.

Immediately after the training, learners completed a Post‐Training Evaluation Survey assessing competence, comfort, and anticipated application of evaluation skills. Ninety days following completion, a 3‐Month Follow‐up Survey assessed perceived skill development and continued application of evaluation practices over time. Survey findings were reviewed to identify strengths, areas for refinement, and opportunities for future iteration (Figure 2).

Surveys were fielded online using the survey platform VA REDCap [12]. The initial Needs Assessment Survey was sent prior to the training, while the Post‐Training Evaluation Survey was delivered immediately following the training and remained open for approximately 1 week. Two reminders were sent to encourage learners to complete the Post‐Training Evaluation Survey.

## 3 Results

### 3.1 EBcT Evaluation Findings: Participants and Recruitment

This manuscript includes findings from two training cohorts of EBcT, which include a total of 20 learners across 9 project teams. Cohort 1 included 4 project teams (8 learners) while Cohort 2 included 5 project teams (12 learners). Project teams ranged from 1 to 8 learners in size. At least 90% of learners were present for each topic session (including didactics and breakout rooms).

EBcT project teams included learners who represented a variety of roles within their project teams, including lead evaluators (7 of 9 project teams, 78%). Project teams included representatives who served as clinical subject matter experts (9 of 20 learners, 45%), project managers (8 of 20 learners, 40%), operational leaders who supported evaluation efforts (7 of 20 learners, 35%), and analysts (2 of 20 learners, 10%). Learners also worked in a variety of roles within VA outside of their evaluation project, including providing clinical care (4 of 20 learners, 20%), providing operational oversight for clinical programs (6 of 20 learners, 30%), and working in positions in research and quality improvement (10 of 20 learners, 50%). The evaluation projects which learners brought into EBcT represented a range of clinical and operational areas, including specialty clinical services (e.g., ophthalmology and rheumatology), hospital‐based care models (e.g., telehospital medicine), workforce and leadership development initiatives (e.g., clinical skills training and educational leadership programs), and staff well‐being.

Both EBcT training cohorts received the same core agenda, materials, and facilitation approach. EBcT project teams were identified through outreach to VA operational offices that actively engage in internal quality improvement projects via collaborations with program evaluation teams. Offices were briefed on the goals, structure, and expectations of the training and were asked to recommend project teams actively working on program evaluation projects which included leadership support; these project teams were viewed as ideal candidates for participation in EBcT. A recruitment flyer outlining prerequisites and participation requirements was also disseminated to multiple operational offices to assist with learner recruitment.

Once identified, project teams determined which staff would attend EBcT. EBcT participation required learners to be actively working on a quality improvement evaluation project within a project team; enrollment by individuals (outside of evaluation project teams) or by teams that were not actively working on a quality improvement evaluation project was not permitted. A project team was defined as a group of learners sharing responsibility for evaluating a specific operational or quality improvement project. This prerequisite ensured that training activities were directly connected to real‐world evaluation efforts and allowed project teams to apply the seven‐step process systematically to their own projects.

### 3.2 EBcT Training Outcomes

Learners indicated a high level of satisfaction throughout the training, with 100% (20 of 20) of respondents indicating they were “Satisfied” or “Very Satisfied” at the end of each training day. Learner attendance was also very high throughout the training, with more than 90% of learners joining all virtual didactic sessions and 100% of learners attending the breakout content application sessions.

A majority (50 + %) of learners indicated that they had achieved levels of self‐perceived skill development and comfort consistent with EBcT's learning targets (assessed separately, learning target based on a 3 or higher on a 1–5 rating scale) for all evaluation skill areas with the exception of “Incorporating Analytic Techniques” (for which 42.1% indicated they had achieved the competency learning target and 50% indicating they had achieved the content comfort target). Respondents also indicated a moderate amount of posttraining application of EBcT skills during the 90‐day period following training completion, with a majority (50 + %) endorsing the use of skills related to “Engaging Partners,” “Developing an Evaluation Plan,” “Gathering Best Evidence,” “Conducting Analyzes,” “Discussing Findings,” “Assessing Changes from Findings,” and “Applying Results for Management.” However, a minority of respondents indicated the application of skills related to “Describing the Program,” “Incorporating Analytic Techniques,” “Identifying Key Outcomes,” “Writing Evaluation Results,” “Developing Recommendations,” and “Assessing Changes from Findings” during the 90‐day post‐training period (Table 1).

**TABLE 1:** Number and proportion of learners who achieved training targets during training and during 90 days posttraining (*N* = 20).

Evaluation skill area	Achieved goal level of skill development1		Achieved goal level of comfort with material2		Skill application during 90 days posttraining3	
	*N*	%	*N*	%	*N*	%
Engaging partners (Step 1)	16	84%	15	79%	6	55%
Describing the program (Step 2)	17	90%	18	95%	5	45%
Developing evaluation plan (Step 3)	14	74%	13	68%	8	73%
Gathering best evidence (Step 4)	13	68%	12	68%	7	64%
Conducting analyzes (Step 5)	11	58%	12	63%	6	55%
Incorporating analytic techniques (Strat Appl)	8	42%	9	50%	4	36%
Identifying key outcomes (Strat Appl)	12	63%	15	79%	5	45%
Writing evaluation results (Strat Appl)	14	74%	14	74%	4	36%
Developing recommendations (Strat Appl)	13	68%	16	84%	3	27%
Discuss fundings/recommendations (Step 6)	18	95%	17	90%	6	55%
Assessing changes from findings (Step 7)	15	79%	14	74%	4	36%
Knowledge of VA expectations (Strat Appl)	14	74%	15	83%	6	55%
Applying results for management (Strat Appl)	16	84%	17	90%	7	64%

*Note:* 1*N* = 19, Skill Development Learning Target based on self‐rated competency score of 3 or higher on 1–5 scale on the Post‐Training Evaluation Survey. 2*N* = 19 for all measures other than Goal Level of Comfort with Material for Evaluation Skill Areas “Gathering Best Evidence,” “Incorporating Analytic Techniques,” and “Knowledge of VA Expectations,” *N* = 18, Comfort Learning Target based on self‐rated comfort score of 3 or higher on 1–5 scale on the Post‐Training Evaluation Survey. 3*N* = 11, Skill Application based on learner yes/no response on the 3‐Month Follow‐Up Survey.

## 4 Discussion

Despite the importance of program evaluation for effective LHSs, opportunities for healthcare professionals to develop applied evaluation skills that support learning cycles remain limited [13]. This results in skill gaps and inconsistent knowledge that may impact the ability of professionals to conduct necessary (and often required) program evaluations. As health systems increasingly adopt LHS principles, the availability of structured, applied training models to build evaluation capacity has become increasingly important. Such training is vital for ensuring that healthcare organizations can engage in program evaluation activities needed to support LHS maturation and effective LHS functioning, such as continuous improvement and optimal patient care [14]. In this manuscript, we highlight the role of Evaluation Bootcamp Training (EBcT) in addressing these needs within the context of VA quality improvement.

EBcT was developed to address a critical gap in program evaluation capacity‐building, providing learners with a comprehensive understanding of evaluation best practices and the practical skills needed for effective application. The training was designed to support professional development, improve access to evaluation resources, and support the creation of a community of learners in ways that support VA's LHS infrastructure. Preliminary outcome information on EBcT suggests that it is feasible for broad VA staff participation (as reflected by strong enrollment and attendance across training sessions), is viewed as relevant and enjoyable (based on high levels of learner satisfaction), and may be able to support the development of applied program evaluation skills (based on a majority of learners indicating that skill targets were met at the end of training). Further, preliminary evidence suggests that learners engaged in evaluation activities during the 90‐day posttraining period and applied skills developed as part of the training.

While training is frequently leveraged to build evaluation capacity [7], training outcomes have largely been assessed within the context of university‐based evaluation training programs [15, 16]. Our work describes an approach for evaluation capacity building through training in a setting that is more directly relevant to the development of functioning LHS. This style of training could be helpful for supporting the maturation of LHSs across different health care settings, as there is a universal need for rigorous evaluation to assess program functioning and determine whether new initiatives were successful [4]. The skills developed as part of EBcT also support the capability of organizations to offer key infrastructural services that characterize an advanced LHS [4].

While the training demonstrated preliminary success in enhancing learner comfort and competency in many program evaluation skill areas, some skills proved challenging to develop within the limited timeframe of EBcT. For example, a lower proportion of learners attained the learning targets related to statistical analysis and data visualization, likely because these activities require specific technical competencies along with extended practice, iterative feedback, and hands‐on application, which were not feasible within the time‐limited structure of EBcT [17]. Similarly, the limited growth observed in areas like Incorporating Analytic Techniques may also be attributed to the technical nature of these skills, which demand real‐world practical experience that cannot be easily incorporated into a brief virtual training [18]. In addition, while most respondents reported engaging in evaluation activities related to evaluation development, management, and interacting with partners during the 90 days following participation in EBcT, fewer indicated that they had participated in activities related to analytic design, measurement, product creation, or the creation of evaluation products. Adding interactive components, structured follow‐up resources, or additional mentorship, which extend training related to these areas could further support skill development in these areas beyond the initial EBcT.

EBcT will continue to be refined based on learner and trainer feedback. For example, training content is being iteratively updated to ensure alignment with current VA guidance on quality improvement project development and execution. Based on learner feedback that it would be helpful to have more time to apply EBcT content between training days, a nonconsecutive three‐day training structure is being explored. Additional content is being added to provide guidance on high‐priority outcomes within VA such as return on investment and the assessment of implementation strategies. Training approaches such as interactive case simulations, which have been shown to deepen learner engagement [19], are being explored for potential inclusion within EBcT. A broader evaluation is being developed to assess the impact of EBcT on post‐training skill application. The goal of this evaluation will be to determine the extent to which EBcT supports the creation of actionable information within projects that later inform operational decision‐making. An additional target of future EBcT evaluation will be the extent to which EBcT training prepares VA employees to more effectively monitor existing programs and implement new programs in their nonevaluation roles, thus supporting VA's ability to rapidly utilize data to inform daily clinical and operational performance. These modifications are intended to further integrate EBcT within VA's broader LHS infrastructure while maintaining its applied, project‐centered design. An additional goal of the refined EBcT model would be the identification of core and adaptable components to support wider adoption in other LHS settings where there is a need to develop additional evaluation capacity.

### 4.1 Limitations

EBcT was intentionally designed as a 4‐day virtual training to balance depth of content with feasibility for VA staff who maintain full‐time operational responsibilities. While this structure increased accessibility across nationally dispersed project teams, it limits the time available for application and refinement, particularly for technically complex skill areas such as statistical analysis and advanced analytic techniques. These skills typically require iterative application and feedback beyond what can be achieved within a condensed format. Furthermore, the training was implemented across two training cohorts representing distinct VA program offices. Although this provided exposure to varied evaluation contexts, the relatively small number of learners and differences in programmatic focus raise questions about the utility of EBcT for a broader VA quality improvement audience. Even so, iterative plans for collecting feedback from learners and updating EBcT content should assist with the refinement of training material based on VA employee needs.

## 5 Conclusion

EBcT addresses a key need within VA by offering opportunities for staff to participate in flexible, guided learning on applied program evaluation best practices based on formal VA guidance. Preliminary information suggests that this training is feasible and acceptable for VA learners and able to support learning related to multiple evaluation skill areas. As such, EBcT has the potential to serve as a key resource in supporting VA as functioning as an LHS. Similar approaches to training could be developed and adopted within other health care settings to support the development of LHS core competencies within staff participating in quality improvement projects.

## Funding

This work was supported by the VA Quality Enhancement Research Initiative (QUERI) (EBP 22‐106). This manuscript represents the views of the authors and does not necessarily represent the views of the Department of Veterans Affairs or the US government.

## Conflicts of Interest

J.P.D. receives personal fees from the American College of Emergency Physicians as a methodology/statistics editor for Annals of Emergency Medicine. The other authors declare no conflicts of interest.

## Supporting information


**Table A1.** The evaluation bootcamp training agenda.

## Data Availability

Research data are not shared.
