# Supplementary material for: Developing a Program Evaluation Training Within a National Federal Healthcare Setting: Veterans Affairs Evaluation Bootcamp Training
Source: Learn Health Syst. 2026 Jul 13;10(3):e70102. doi: 10.1002/lrh2.70102 (PMC13364505; doi:10.1002/lrh2.70102)
Supplement: Supplementary file 1 — Table A1. The evaluation bootcamp training agenda. [file LRH2-10-e70102-s001.docx]

**Supplement 1**

**Relevant Questions from Needs Assessment Survey**

| **Competence and Comfort** |
| --- |
| The following tasks below are related to program evaluation best practices as presented in the QUERI Evaluation Guide.    In the table below, please assess your level of Competence in the following tasks related to program evaluation with 1 being "No Knowledge" to 5 being an "Expert". Competence is the quality or state of having sufficient knowledge, judgment, skill, or strength (as for a particular duty or in a particular respect).    In the same table below, please assess your Comfort level in performing the following tasks related to program evaluation with 1 being "Not at all comfortable" to 5 being "Very comfortable". Comfort is the ease or alleviation of a person's feelings of distress in completing a task.     \| **Tasks Related to Evaluation** \| **Competence** \| **Comfort** \| \| --- \| --- \| --- \| \| **1 = No Knowledge** \| **1 = Not at all comfortable** \| \| **5 = Expert** \| **5 = Very comfortable** \| \| Engaging partners (stakeholders) in your evaluation project \| \| 1 \| 2 \| 3 \| 4 \| 5 \| \| --- \| --- \| --- \| --- \| --- \| \| \| 1 \| 2 \| 3 \| 4 \| 5 \| \| --- \| --- \| --- \| --- \| --- \| \| \| Describing the program and problem \| \| 1 \| 2 \| 3 \| 4 \| 5 \| \| --- \| --- \| --- \| --- \| --- \| \| \| 1 \| 2 \| 3 \| 4 \| 5 \| \| --- \| --- \| --- \| --- \| --- \| \| \| Developing an evaluation plan \| \| 1 \| 2 \| 3 \| 4 \| 5 \| \| --- \| --- \| --- \| --- \| --- \| \| \| 1 \| 2 \| 3 \| 4 \| 5 \| \| --- \| --- \| --- \| --- \| --- \| \| \| Gathering the best available evidence to assess your evaluation questions \| \| 1 \| 2 \| 3 \| 4 \| 5 \| \| --- \| --- \| --- \| --- \| --- \| \| \| 1 \| 2 \| 3 \| 4 \| 5 \| \| --- \| --- \| --- \| --- \| --- \| \| \| Conducting analyses and forming conclusions \| \| 1 \| 2 \| 3 \| 4 \| 5 \| \| --- \| --- \| --- \| --- \| --- \| \| \| 1 \| 2 \| 3 \| 4 \| 5 \| \| --- \| --- \| --- \| --- \| --- \| \| \| Incorporating analytic techniques used in your evaluation (e.g., clinical significance) \| \| 1 \| 2 \| 3 \| 4 \| 5 \| \| --- \| --- \| --- \| --- \| --- \| \| \| 1 \| 2 \| 3 \| 4 \| 5 \| \| --- \| --- \| --- \| --- \| --- \| \| \| Identifying operationalized key outcomes based on data \| \| 1 \| 2 \| 3 \| 4 \| 5 \| \| --- \| --- \| --- \| --- \| --- \| \| \| 1 \| 2 \| 3 \| 4 \| 5 \| \| --- \| --- \| --- \| --- \| --- \| \| \| Writing your evaluation results \| \| 1 \| 2 \| 3 \| 4 \| 5 \| \| --- \| --- \| --- \| --- \| --- \| \| \| 1 \| 2 \| 3 \| 4 \| 5 \| \| --- \| --- \| --- \| --- \| --- \| \| \| Developing recommendations based on evaluation finding \| \| 1 \| 2 \| 3 \| 4 \| 5 \| \| --- \| --- \| --- \| --- \| --- \| \| \| 1 \| 2 \| 3 \| 4 \| 5 \| \| --- \| --- \| --- \| --- \| --- \| \| \| Discussing findings and recommendations with partners (stakeholders) \| \| 1 \| 2 \| 3 \| 4 \| 5 \| \| --- \| --- \| --- \| --- \| --- \| \| \| 1 \| 2 \| 3 \| 4 \| 5 \| \| --- \| --- \| --- \| --- \| --- \| \| \| Assessing changes from evaluation findings \| \| 1 \| 2 \| 3 \| 4 \| 5 \| \| --- \| --- \| --- \| --- \| --- \| \| \| 1 \| 2 \| 3 \| 4 \| 5 \| \| --- \| --- \| --- \| --- \| --- \| \| \| Knowledge of VA expectations and guidelines for program evaluation \| \| 1 \| 2 \| 3 \| 4 \| 5 \| \| --- \| --- \| --- \| --- \| --- \| \| \| 1 \| 2 \| 3 \| 4 \| 5 \| \| --- \| --- \| --- \| --- \| --- \| \| \| Applying the results of a program evaluation to inform plans for program management/adaptation \| \| 1 \| 2 \| 3 \| 4 \| 5 \| \| --- \| --- \| --- \| --- \| --- \| \| \| 1 \| 2 \| 3 \| 4 \| 5 \| \| --- \| --- \| --- \| --- \| --- \| \| |

**Supplement 2**

**Relevant Questions from Evaluation (Post-Training) Survey**

| **Satisfaction** |
| --- |
| How satisfied are you with the Evaluation Bootcamp Training? |
| - Very Satisfied - Satisfied - Unsatisfied - Very Unsatisfied |

| **Competence and Comfort** |
| --- |
| The following tasks below are related to program evaluation best practices as presented in the QUERI Evaluation Guide.  Now that you have participated in the Evaluation Bootcamp Training, please assess your level of Competence in the following tasks related to program evaluation with 1 being "No Knowledge" to 5 being an "Expert" in the table below. Competence is the quality or state of having sufficient knowledge, judgment, skill, or strength (as for a particular duty or in a particular respect).  In the same table below, please assess your Comfort level in performing the following tasks related to program evaluation with 1 being "Not at all comfortable" to 5 being "Very comfortable". Comfort is the ease or alleviation of a person's feelings of distress in completing a task.     \| **Tasks Related to Evaluation** \| **Competence** \| **Comfort** \| \| --- \| --- \| --- \| \| **1 = No Knowledge** \| **1 = Not at all comfortable** \| \| **5 = Expert** \| **5 = Very comfortable** \| \| Engaging partners (stakeholders) in your evaluation project \| \| 1 \| 2 \| 3 \| 4 \| 5 \| \| --- \| --- \| --- \| --- \| --- \| \| \| 1 \| 2 \| 3 \| 4 \| 5 \| \| --- \| --- \| --- \| --- \| --- \| \| \| Describing the program and problem \| \| 1 \| 2 \| 3 \| 4 \| 5 \| \| --- \| --- \| --- \| --- \| --- \| \| \| 1 \| 2 \| 3 \| 4 \| 5 \| \| --- \| --- \| --- \| --- \| --- \| \| \| Developing an evaluation plan \| \| 1 \| 2 \| 3 \| 4 \| 5 \| \| --- \| --- \| --- \| --- \| --- \| \| \| 1 \| 2 \| 3 \| 4 \| 5 \| \| --- \| --- \| --- \| --- \| --- \| \| \| Gathering the best available evidence to assess your evaluation questions \| \| 1 \| 2 \| 3 \| 4 \| 5 \| \| --- \| --- \| --- \| --- \| --- \| \| \| 1 \| 2 \| 3 \| 4 \| 5 \| \| --- \| --- \| --- \| --- \| --- \| \| \| Conducting analyses and forming conclusions \| \| 1 \| 2 \| 3 \| 4 \| 5 \| \| --- \| --- \| --- \| --- \| --- \| \| \| 1 \| 2 \| 3 \| 4 \| 5 \| \| --- \| --- \| --- \| --- \| --- \| \| \| Incorporating analytic techniques used in your evaluation (e.g., clinical significance) \| \| 1 \| 2 \| 3 \| 4 \| 5 \| \| --- \| --- \| --- \| --- \| --- \| \| \| 1 \| 2 \| 3 \| 4 \| 5 \| \| --- \| --- \| --- \| --- \| --- \| \| \| Identifying operationalized key outcomes based on data \| \| 1 \| 2 \| 3 \| 4 \| 5 \| \| --- \| --- \| --- \| --- \| --- \| \| \| 1 \| 2 \| 3 \| 4 \| 5 \| \| --- \| --- \| --- \| --- \| --- \| \| \| Writing your evaluation results \| \| 1 \| 2 \| 3 \| 4 \| 5 \| \| --- \| --- \| --- \| --- \| --- \| \| \| 1 \| 2 \| 3 \| 4 \| 5 \| \| --- \| --- \| --- \| --- \| --- \| \| \| Developing recommendations based on evaluation finding \| \| 1 \| 2 \| 3 \| 4 \| 5 \| \| --- \| --- \| --- \| --- \| --- \| \| \| 1 \| 2 \| 3 \| 4 \| 5 \| \| --- \| --- \| --- \| --- \| --- \| \| \| Discussing findings and recommendations with partners (stakeholders) \| \| 1 \| 2 \| 3 \| 4 \| 5 \| \| --- \| --- \| --- \| --- \| --- \| \| \| 1 \| 2 \| 3 \| 4 \| 5 \| \| --- \| --- \| --- \| --- \| --- \| \| \| Assessing changes from evaluation findings \| \| 1 \| 2 \| 3 \| 4 \| 5 \| \| --- \| --- \| --- \| --- \| --- \| \| \| 1 \| 2 \| 3 \| 4 \| 5 \| \| --- \| --- \| --- \| --- \| --- \| \| \| Knowledge of VA expectations and guidelines for program evaluation \| \| 1 \| 2 \| 3 \| 4 \| 5 \| \| --- \| --- \| --- \| --- \| --- \| \| \| 1 \| 2 \| 3 \| 4 \| 5 \| \| --- \| --- \| --- \| --- \| --- \| \| \| Applying the results of a program evaluation to inform plans for program management/adaptation \| \| 1 \| 2 \| 3 \| 4 \| 5 \| \| --- \| --- \| --- \| --- \| --- \| \| \| 1 \| 2 \| 3 \| 4 \| 5 \| \| --- \| --- \| --- \| --- \| --- \| \| |

**Supplement 3**

**Relevant Questions from 90 Day/3 Month Follow-Up Survey**

| **Benefits Gained** |
| --- |
| Which of these activities have you participated in as a result of the benefits gained from the Evaluation Bootcamp Training? (Check all that apply) |
| - Engaging partners (stakeholders) in your evaluation project - Describing the program and problem - Further expanding on your evaluation plan - Gathering the best available evidence to assess your evaluation questions - Conducting analyses and forming conclusions - Incorporating analytic techniques used in your evaluation - Identifying operationalized key outcomes based on data - Writing evaluation results - Developing recommendations based on evaluation findings - Discussing findings and recommendations with partners - Assessing changes from evaluation findings - Using knowledge of VA expectations and guidelines for program evaluation - Applying the results of a program evaluation to inform plans for program management/adaptation |

**Appendix**

**Table A1**

**The Evaluation Bootcamp Training Agenda**

| **Module/Breakout Room** | **Day** | **Time** |
| --- | --- | --- |
| Evaluation Basics in VA (Strategic Application) | 1 | 50 min |
| Logic Modeling (Strategic Application) |  | 20 min |
| Breakout Room: Logic Modeling Exercise |  | 40 min |
| Optional Office Hours |  | 1 hour |
| Engage Stakeholders (Step 1) | 2 | 30 min |
| Describe the Problem (Step 2) |  |  |
| Breakout Room: Step 1,2 |  | 1 hour |
| Develop an Evaluation Plan (Step 3) |  | 1 hour 25 mins |
| Breakout Room: Step 3 |  | 1 hour |
| Optional Office Hours |  | 1 hour |
| Gather Best Evidence (Step 4) | 3 | 45 min |
| Conduct Analyses and Form Conclusions (Step 5) |  |  |
| Breakout Room: Step 4/5 |  | 1 hour |
| Discuss findings and Recommendations with Stakeholders (Step 6) |  | 45 min |
| Assess Changes from Initial Findings (Step 7) |  |  |
| Breakout Room: Step 6, 7 |  | 1 hour |
| Optional Office Hours |  | 1 hour |
| Evaluation Management (Strategic Application) | 4 | 40 min |
| Breakout Room: Evaluation Management and Strategic Application |  | 30 min |
| Evaluation Resources (Strategic Application) |  | 30 min |
| Office Hours to prepare for Evaluation Plan Presentation |  | 1 hour |
| Evaluation Plan Presentations |  | 1 hour 15 min |
